# Mice left out in the cold: commentary on the phenotype of TRPM8-nulls

**DOI:** 10.1186/1744-8069-3-23

**Published:** 2007-08-17

**Authors:** Richard L Daniels, David D McKemy

**Affiliations:** 1 Neuroscience Graduate Program, Department of Biological Sciences, University of Southern California, Los Angeles, CA90089-0641, USA; 2 Neuroscience Graduate Program, Department of Biological Sciences, and School of Dentistry, University of Southern California, Los Angeles, CA90089-0641, USA

## Abstract

Detection of innocuous temperatures allows an organism to select an appropriate environmental climate, while the ability to recognize noxious temperature extremes warns of impending tissue damage. For temperatures considered cold, the menthol receptor TRPM8 is activated when temperatures drop below ~26°C, thus making it an intriguing candidate as the molecular mediator of cold perception. However, confirmation of this hypothesis in vivo has eluded researchers until recently. Three independent research groups have reported that mice lacking this single gene are severely impaired in their ability to detect cold temperatures. Remarkably, these animals are deficient in many diverse aspects of cold signaling, including cool and noxious cold perception, injury-evoked sensitization to cold, and cooling-induced analgesia. These animals provide a great deal of insight into the molecular signaling pathways that participate in the detection of cold and painful stimuli.

## Background

The ability of all species to detect ever-changing environmental temperatures is critical for homeostasis and survival. Recently, three research groups, led by Bautista, Colburn, and Dhaka, have reported that mice with a disruption in the gene encoding the cold and menthol receptor *TRPM8 *exhibit remarkable deficiencies in a range of cold responses [1-3]. These results suggest that TRPM8 is the predominant detector of cold temperatures in vivo, and serves a number of important roles in somatosensation, nociception, and analgesia.

The last decade has yielded remarkable advances in our understanding of the molecular basis of thermosensation and pain (nociception), particularly with the cloning of a number of temperature-gated, excitatory ion channels of the Transient Receptor Potential (TRP) family. The founding member of this family, TRPV1, is a polymodal receptor that detects noxious heat (>43°C), acidity, and capsaicin, the 'hot' component in chili peppers [4]. Subsequently, other members of the TRPV subfamily were shown to be gated by heat and warmth, including TRPV2, TRPV3, and TRPV4 [5]. Mice lacking each of these channels (except for TRPV2) have been generated, and all show profound deficits in thermosensory behaviors consistent with their roles as molecular thermosensors.

While heat directly gates many TRPV channels, cold temperatures have been reported to activate two additional TRP channels. TRPA1 was initially reported to be activated by noxious cold (<17°C), but these results have been controversial [6]. More recent studies have shown TRPA1 to be activated by a myriad of agonists, including certain environmental irritants, pungent compounds such as mustard oil, cinnamon, and garlic, and increased intracellular calcium [7,8]. Studies of TRPA1-null mice have been less than definitive regarding the in vivo role of this channel in thermosensation. While two independent strains have confirmed the channel's chemosensory role, the researchers disagree as to whether TRPA1 is involved in thermosensation [9,10]. One study reported cold-sensitivity in TRPA1-nulls to be indistinguishable from wild-type mice, whereas the other described deficits in noxious cold sensing. To further complicate matters, deficits were only significant in TRPA1-null females. Thus, it remains to be seen whether or not TRPA1 functions as a cold sensor in vivo.

In contrast to TRPA1, the cold-sensitivity of TRPM8 is well-established. In vitro, the channel is a receptor for a number of compounds which evoke the psychophysical sensation of cold (such as menthol and icilin), and is activated by temperatures that range from innocuous cool (26–15°C) to noxious cold (<15°C). While these in vitro data suggest a definitive role for TRPM8 in cold sensation, several key questions regarding its in vivo role have remained. Is TRPM8 a physiological transducer of innocuous cool and/or noxious cold? Is TRPM8 involved in the hypersensitivity to cold caused by inflammation or nerve injury? Is TRPM8 responsible for the analgesic effect produced by cold or chemical cooling compounds? Many of these questions have been addressed in three recent reports of the behavioral phenotype of TRPM8 knock out mice, and have provided a more accurate description of the physiological underpinnings of cold sensation.

## Is TRPM8 a physiological transducer of innocuous cool and/or noxious cold?

TRPM8 becomes active at temperatures below 26°C in vitro, and is therefore thought to mediate the sensation of innocuous cool. However, the steep temperature-dependence of TRPM8 currents also extends its activity into the noxious range, reported to begin at temperatures lower than 15°C in psychophysical studies [6]. Thus, it is not clear if TRPM8 serves to detect innocuous cool, noxious cold, or both. To address this, all three groups performed temperature preference assays with TRPM8-nulls, presenting mice with a choice between two temperature zones. Wild-type mice prefer to remain within a relatively narrow temperature range (between 30–38°C), avoiding cooler temperatures. However, mice lacking TRPM8 no longer display this avoidance, and spend approximately equal times in both warm and cool thermal zones. Although all three studies report that TRPM8-nulls are deficient in avoiding certain temperatures, discrepancies exist as to the exact temperature ranges affected. Bautista et al. report that TRPM8-nulls regain aversion to cold temperatures at or below 10°C, while Colburn et al. observe that their knockout mice do not avoid temperatures as cold as 5°C. Moreover, in cold plate assays, Colburn et al. report that TRPM8-nulls have longer paw withdrawal latencies at 0°C than their wild-type littermates. In contrast, Bautista et al. and Dhaka et al. found no differences in behavioral responses under similar experimental conditions. Of note, each study reported dramatically different absolute latencies. For example, the time to paw withdrawal at 0°C (-1°C in Dhaka et al.) in wild-type mice ranged from 5–50 seconds between the three studies. These significant differences in animal behavior highlight the difficulty of these assays, and suggest that some of the discrepancies between the various reports of TRPM8-nulls may be due to differences in experimental paradigms. Nonetheless, these results establish the necessity of TRPM8 in thermosensation, and demonstrate that in vivo, the channel mediates the detection of innocuous cool and perhaps a component of noxious cold.

While each study reported that TRPM8-null mice have severe thermosensory deficits, they nevertheless retained an aversion to very cold temperatures, as stated above. In addition, all three studies reported a small residual population of neurons from TRPM8-null animals that respond to cold in culture with an increase in intracellular calcium. Alternative mechanisms have been proposed for cold-sensing, including temperature-independent responses to cold-induced tissue damage, changes in vascular tone during cold temperatures, and inhibition of warm-sensitive fibers [11]. But while these explanations remain plausible in vivo, they do not explain the functional data from neuronal cultures that demonstrate cold responses even in single neurons lacking TRPM8. Differences in experimental protocols make the exact results difficult to compare, but data from Bautista et al. and Dhaka et al. suggest that in neuronal cultures, a deep cooling response begins around the "noxious threshold" of 12–15°C. Bautista et al. extend these results with recordings from intact fibers in a skin-nerve preparation. Consistent with their culture experiments, fewer cold-sensitive afferents respond to cold (4% in TRPM8-null mice as compared with 16.7% in wild-type). Thus it seems likely that this TRPM8-independent cold-responsiveness is mediated by other molecular pathways. This begs the question: which molecules could be mediating the residual cold response in TRPM8-null cells? There are several possibilities. TRPA1 remains a candidate, despite conflicting data from behavioral studies of TRPA1-null mice. Several groups have postulated that TRPA1 acts in a receptor-operated manner [12] and is gated by increased intracellular calcium [7,13]. Under this premise, activation of the channel by cold in heterologous cell types would arise indirectly from cold-induced increases in intracellular calcium. Another line of evidence challenges the assumption that TRPA1 is a physiological cold-sensor, namely that cold responsive, menthol insensitive neurons do not respond to any of the known TRPA1 agonists [14]. However, to further complicate the issue, a recent biophysical study utilizing single channel recordings did report direct gating of the channel by cold bath temperatures [15].

Other ion channels have been proposed to contribute to the cold-sensing in some populations of thermosensitive neurons as well. Viana et al. have described a temperature-dependence of certain potassium conductances which would support this hypothesis, suggesting a scenario where cold temperatures increase the excitability of a neuron by keeping it moderately depolarized [16]. In addition, a recent study shows that cold temperatures decrease the activation threshold of Na_V_1.8, a voltage-gated sodium channel expressed in a subset of nociceptive sensory neurons [17]. In the same way, this could lead to increased nociceptor excitability at cold temperatures.

## What is the role of TRPM8 in injury-evoked hypersensitivity and cooling-induced analgesia?

Inflammation greatly impacts thermosensation, resulting in heightened sensitivity to temperatures in the innocuous range (allodynia), and in the already painful range (hyperalgesia). This sensitization of nociceptors is thought to be caused by proalgesic components of the "inflammatory soup", including molecules such as bradykinin and prostaglandins [5,18]. However, studies by Reid et al. have demonstrated that proalgesic agents such as bradykinin desensitize menthol-sensitive neurons, a seemingly contradictory finding [19]. Colburn et al. used two different models to assess the involvement of TRPM8 in injury-evoked hypersensitivity to cold. In a model of neuropathic pain where the sciatic nerve is chronically irritated, wild-type animals shake their hind legs in response to the evaporative cooling caused by acetone application. Injured TRPM8-null mice, however, display no such nocifensive behaviors, similar to uninjured control animals. They obtained similar results when using the CFA model of inflammatory injury. These results suggest that TRPM8 may mediate the majority of cold allodynia and hyperalgesia, though using cold as the stimulus (rather than evaporative cooling) will validate these results further.

In a fascinating study, Proudfoot et al. demonstrated that topical application of cold or cooling compounds produces a temporary analgesic effect mediated by TRPM8-expressing afferents [20]. Using a rodent model of neuropathic pain which measured paw withdrawal latencies in response to mechanical or thermal stimuli, the investigators observed longer paw withdrawal latencies in animals first treated with cold or cooling compounds. In a similar manner, they found that pain behaviors were reduced in several models of inflammatory pain. This analgesia persists for 20–40 minutes, at which point the animals behave similar to those not pre-treated with cold or cooling-compounds. Dhaka et al. examined whether this same analgesic effect is present in TRPM8-nulls using formalin (a compound that evokes acute pain followed by inflammation), injected into the hindpaws of mice. The analgesic effects of cold were then assessed by placing mice on plates set to 17°C or 24°C. Wild-type mice show marked decreases in pain behaviors (licking and lifting hindpaws) during the acute pain phase while standing on a plate set to 17°C. However, mice lacking TRPM8 behaved similarly to wild-type animals that did not have the benefit of a cool (24°C) surface. Together with the results of Proudfoot et al., these data indicate that TRPM8 is mediating analgesia in early phases of the inflammatory pain response and in chronic neuropathic pain as well. In addition, these observations suggest a way to reconcile the apparent contradiction in Reid's observations of TRPM8-expressing neuronal desensitization in response to proalgesic agents. Inflammation may, in desensitizing TRPM8-neurons, lead to hypersensitivity by inhibiting the otherwise analgesic activity of cold temperatures.

## Conclusion

These elegant studies have each provided a powerful tool for the continued study of thermosensation and pain, yet several important questions remain to be addressed. Is TRPA1 involved in thermosensation? One hopes that behavioral responses from mice lacking both TRPM8 and TRPA1 will demonstrate whether TRPA1 is involved in noxious cold sensing. How do TRPM8-expressing neurons mediate analgesia? It will be important to continue investigating TRPM8's role in cooling-induced analgesia. It will be valuable to uncover the central mechanisms that cause this analgesia, and to differentiate between the neural mechanisms that govern each phase of the pain response. Could it be that there are multiple populations of TRPM8-expressing afferents, with some mediating analgesia and some mediating cold hypersensitivity? Finally, it will be fascinating to learn what, if any, role TRPM8 plays in other physiological processes, as it is not only localized in sensory neurons but has also been found to be expressed in other tissue types. With regard to the present studies, each has provided strong evidence that TRPM8 is the primary transducer of cool temperatures in mammals. Future studies that utilize these knockout animals will surely complete the picture of the molecular signal transduction pathways essential for thermosensation, and in turn provide a more complete description of how these same molecules contribute to pain at its most fundamental level.
